# Effectiveness of Alberta Family Integrated Care on infant length of stay in level II neonatal intensive care units: a cluster randomized controlled trial

**DOI:** 10.1186/s12887-020-02438-6

**Published:** 2020-11-28

**Authors:** Karen M. Benzies, Khalid Aziz, Vibhuti Shah, Peter Faris, Wanrudee Isaranuwatchai, Jeanne Scotland, Jill Larocque, Kelly J. Mrklas, Christopher Naugler, H. Thomas Stelfox, Radha Chari, Amuchou Singh Soraisham, Albert Richard Akierman, Ernest Phillipos, Harish Amin, Jeffrey S. Hoch, Pilar Zanoni, Jana Kurilova, Abhay Lodha, the Alberta FICare Level II NICU Study Team

**Affiliations:** 1 Faculty of Nursing, University of Calgary, Calgary, AB T2N 1N4 Canada; 2 Cumming School of Medicine, University of Calgary, Calgary, AB Canada; 3 Faculty of Medicine and Dentistry, University of Alberta, Edmonton, AB Canada; 4 Northern Alberta Neonatal Program, Stollery Children’s Hospital, Edmonton, AB Canada; 5 Faculty of Medicine, University of Toronto, and Mount Sinai Hospital, Toronto, ON Canada; 6 Analytics, Data Integration, Measurement & Reporting, Alberta Health Services, Calgary, AB Canada; 7 Institute of Health Policy, Management and Evaluation, University of Toronto, Toronto, ON Canada; 8 Neonatal Intensive Care Unit, Rockyview General Hospital, Alberta Health Services, Calgary, AB Canada; 9 Strategic Clinical Networks™, System Innovation and Programs, Alberta Health Services, Calgary, AB Canada; 10 Department of Public Health Sciences, University of California Davis, Davis, CA USA; 11 Center for Healthcare Policy and Research, University of California Davis, Sacramento, CA USA

**Keywords:** Family integrated care, Preterm infant, Neonatal intensive care unit, Length of stay, Family centered care, Bundled model of care, Relational communication, Parent education, Parent support, Health services research

## Abstract

**Background:**

Parents of infants in neonatal intensive care units (NICUs) are often unintentionally marginalized in pursuit of optimal clinical care. Family Integrated Care (FICare) was developed to support families as part of their infants’ care team in level III NICUs. We adapted the model for level II NICUs in Alberta, Canada, and evaluated whether the new Alberta FICare™ model decreased hospital length of stay (LOS) in preterm infants without concomitant increases in readmissions and emergency department visits.

**Methods:**

In this pragmatic cluster randomized controlled trial conducted between December 15, 2015 and July 28, 2018, 10 level II NICUs were randomized to provide Alberta FICare™ (*n* = 5) or standard care (*n* = 5). Alberta FICare™ is a psychoeducational intervention with 3 components: Relational Communication, Parent Education, and Parent Support. We enrolled mothers and their singleton or twin infants born between 32 ^0/7^ and 34 ^6/7^ weeks gestation. The primary outcome was infant hospital LOS. We used a linear regression model to conduct weighted site-level analysis comparing adjusted mean LOS between groups, accounting for site geographic area (urban/regional) and infant risk factors. Secondary outcomes included proportions of infants with readmissions and emergency department visits to 2 months corrected age, type of feeding at discharge, and maternal psychosocial distress and parenting self-efficacy at discharge.

**Results:**

We enrolled 654 mothers and 765 infants (543 singletons/111 twin cases). Intention to treat analysis included 353 infants/308 mothers in the Alberta FICare™ group and 365 infants/306 mothers in the standard care group. The unadjusted difference between groups in infant hospital LOS (1.96 days) was not statistically significant. Accounting for site geographic area and infant risk factors, infant hospital LOS was 2.55 days shorter (95% CI, − 4.44 to − 0.66) in the Alberta FICare™ group than standard care group, *P* = .02. Secondary outcomes were not significantly different between groups.

**Conclusions:**

Alberta FICare™ is effective in reducing preterm infant LOS in level II NICUs, without concomitant increases in readmissions or emergency department visits. A small number of sites in a single jurisdiction and select group infants limit generalizability of findings.

**Trial registration:**

ClinicalTrials.gov Identifier NCT02879799, retrospectively registered August 26, 2016.

## Introduction

Globally, the estimated rate of preterm birth is 10.6% (regional range 8.7–13.4%) [1]. The preterm birth rate in Alberta, 9.2% in 2018–2019, is the highest among Canadian provinces [2]. Approximately 15% of preterm infants are born at < 32 weeks gestational age (GA) [1] and require care in a level III neonatal intensive care unit (NICU) [3],while 85% are born between 32 and 36 weeks GA [1] and may require care in a level II NICU [3]. The unexpectedness of preterm birth leaves parents feeling overwhelmed, anxious, depressed, isolated, and unprepared to interact with, and care for, their infant [4–6]. Preterm birth and experiences in the NICU disrupt the early parent-infant relationship, which is critical for brain and biological development [7–9]. In pursuit of optimal infant care, parents are often unintentionally marginalized [10–12]. Models that integrate families into the care team show promise, but lack well-defined components, theoretically driven underpinnings, and contextualization for levels of NICU care.

Building on a foundational clinical program in Estonia [13, 14], Family Integrated Care (FICare) is a model of care developed to involve and support families as part of their infants’ care team in level III NICUs [15, 16]. In a pilot study, compared to a retrospectively matched control group (*n* = 62), at 21 days post-enrolment infants receiving FICare (*n* = 31) showed an increased rate of weight gain and breastfeeding, while mothers reported less stress [15]. In an international cluster randomized controlled trial (cRCT) of FICare in level III NICUs, compared to the standard care group (*n* = 891), at 21 days post-enrolment infants in the FICare group (*n* = 895) showed significantly increased weight gain trajectory and high frequency breastmilk feeding (> 6 times per day), and mothers reported less stress and anxiety [16].

With these positive findings, there was momentum to implement level III FICare in level II NICUs in Alberta. To address concerns about goodness of fit from provincial stakeholders, including nurses, allied health professionals, physicians, hospital administrators, parents, and researchers, we adapted the level III model to the level II NICU setting in Alberta and conducted a cRCT. The aim of the trial was to evaluate the clinical effectiveness and costs of Alberta FICare™ [17]. In this paper, we report the primary outcome, infant hospital length of stay (LOS), and the secondary outcomes of hospital readmissions and emergency department visits to 2 months corrected age (CA), feeding type at discharge, and maternal psychosocial distress and parenting self-efficacy at discharge. We hypothesized that compared to standard care, Alberta FICare™ would (1) decrease infant hospital LOS by 10%, or 1.6 days, without a concomitant increase in hospital readmissions and emergency department visits, and (2) increase rates of breastmilk feeding, reduce maternal psychosocial distress, and increase parenting self-efficacy at discharge.

## Methods

### Study design

We conducted this pragmatic cRCT (ClinicalTrials.gov: NCT02879799) in 10 level II NICUs across 6 cities in Alberta, Canada [17]. All 10 level II NICUs in the province were eligible and agreed to participate. Alberta’s single, publicly funded health care system offers advantages for multicentre studies in terms of standardisation of many structures and processes. Family Centred Care (FCC) is the currently accepted philosophy of care; however, staff do not receive training in the principles of FCC, nor had any sites implemented FICare previously. Site-level administrative endorsement facilitated unit-level changes to care at intervention sites. We did not establish stopping guidelines or a data monitoring committee because Alberta FICare™ was expected to enhance standard care and not cause harm. Multijurisdictional ethical approval was obtained from University of Calgary, Conjoint Health Research Ethics Board (ID 15–0067), University of Alberta, Health Research Ethics Board (Pro00060324), and Covenant Health, Health Research Ethics Board (ID 1762).

### Participants

We included mothers and their preterm singleton or twin infants born between 32^0/7^ and 34^6/7^ weeks gestational age (GA) inclusive, with a primary admission or transfer within 72 h to a level II NICU. If otherwise healthy, preterm infants are typically discharged from hospital at approximately 36^0/7^ weeks. Thus, we capped GA at 34^6/7^ to ensure a one-week minimum exposure to the intervention. We excluded mothers (1) whose health, social, or language issues may have interfered with their ability to communicate with the health care team, (2) with triplets or higher order multiple births, and (3) whose infants required palliative care or had severe congenital or chromosomal anomalies. At intervention sites, we included mothers who committed to spending a minimum of 6 h per day with their infant(s).

### Randomization and masking

The biostatistician stratified hospitals by size (*n* = 6 larger, urban sites; *n* = 4 smaller, regional sites) based on number of infants admitted per year and randomly assigned sites to group: Alberta FICare™ (intervention, *n* = 5) or standard care (*n* = 5). Simple random sampling within stratum resulted in equal numbers of urban and regional sites in each group. The pragmatic nature of the intervention precluded masking of participants and health care providers (HCP).

### Intervention

The goal of Alberta FICare™ is change in culture and practice that involves and supports parents in their role while their infant is receiving care in a level II NICU. Alberta FICare™ is a theoretically driven, dynamic, psychoeducational model of care that empowers parents to build their knowledge, skill, and confidence in caring for their infant(s) in the NICU to prepare for earlier discharge. The model has 3 main components: Relational Communication, Parent Education, and Parent Support. Relational Communication is based on family systems theory [18] and uses circular pattern diagrams and questioning practices to negotiate parent and HCP roles as parents gain confidence in providing care [19]. As partners in the health care team, parents are encouraged, as they are ready and willing, to introduce and share information about their infant during bedside rounds, and empowered to participate in decision-making and care planning. Commendations are used as positive feedback to parents [19]. Parent Education is based on adult learning [20] and self-efficacy theories [21], and includes a Parent Education Pathway, individual bedside teaching, group parent education sessions, and Life’s Little Love app (© 2015 Alexiatek). Multimodal evidence-informed educational approaches provide consistency across HCP. Parent Support is based on stress and coping theory [22]. Professional support includes postpartum depression screening and referrals to ensure mothers receive timely psychological support. Family Mentors, parents who had experience with a preterm infant in the NICU, provide peer-to-peer support. Families in the intervention group received a parking pass.

Infants and mothers at standard care sites received NICU care as usual, with the addition of postpartum depression screening and referral to additional supports if needed. Families in both groups received an investigator-designed parent journal in which they could write about their experiences and keep a daily record of time spent in NICU. On average, families in both groups spent more than 6 h per day in the NICU (9.00 h [*SD* = 5.35] in the Alberta FICare™ group vs 7.79 h [*SD* = 4.87] in the standard care group; *t*(379) = 2.221, *P* = 0.03). The intervention group journal had additional pages for parents to record daily updates on infant weight gain/loss and feeding, questions for the care team, and participation in educational and support activities.

### Procedures

For all managers and Super-Users (specially trained nurses), we provided 4 h of training about the purpose of the study, participant screening and informed consent, and data collection. Super-Users at intervention sites received an additional 8 h of training, including tools and strategies to incorporate Alberta FICare™ into daily practice, and then trained staff at their site. The Project Coordinator completed quarterly site visits to (1) liaise with Super-Users regarding recruitment and data collection, (2) deliver booster doses of training to Super-Users and HCP, as needed, and (3) complete fidelity audits using an investigator-designed checklist. Preliminary analysis of fidelity data shows that Alberta FICare™ was delivered with 71% fidelity at 4 of 5 intervention sites; the fifth site was a regional site that struggled with physician coverage for bedside rounds, a population at higher social risk, and constrained hospital resources including high staff turnover. Although not trained to do so, some standard care sites claimed to be practicing Alberta FICare™. We observed that some standard care sites demonstrated more family centred practices such as enabling parental participation in bedside rounds, which was likely due to diffusion of innovation. Our assessment of fidelity suggested only 30% compliance with Alberta FICare™ at standard care sites.

Super-Users used a standardized script to screen and inform potential participants about the study, answered questions, and obtained written informed consent. Mothers completed a baseline survey as soon as they were well enough, within 72 h of their infant(s) being admitted, and a discharge survey in the 72 h prior to their infant(s) being discharged home. Super-Users collected data from infants’ medical records following discharge. We obtained data on readmissions and emergency department visits from an administrative database.

### Outcomes

The primary outcome was infant hospital LOS, measured from date and time of birth to date and time of discharge. Secondary infant outcomes were proportion of infants requiring hospital readmission to 2 months CA, proportion of infants with an emergency department visit to 2 months CA, and type of feeding at discharge (breastmilk only, combination of breastmilk and formula, or formula only). Secondary maternal outcomes were psychosocial distress (anxiety, depressive symptoms, and stress) and parenting self-efficacy at discharge. Using validated self-report scales, we measured anxiety using the State-Trait Anxiety Inventory (STAI) [23], depressive symptoms using the Edinburgh Postnatal Depression Scale (EPDS) [24], stress using the Parental Stressor Scale: NICU (PSS:NICU) [25], and parenting self-efficacy using the Perceived Maternal Parenting Self-Efficacy (PMP S-E) tool [26]. See Table 1 for descriptions of the measures.

**Table 1:** Maternal outcome measures

Measure	Description
State-Trait Anxiety Inventory (STAI) [22]	40-item instrument that measures the presence and severity of current anxiety symptoms (State Anxiety subscale, 20 items) as well as a generalized, relatively stable tendency to be anxious (Trait Anxiety subscale, 20 items). The theoretical range of subscale scores is 20 to 80, with higher scores indicating greater anxiety. Internal consistency (0.86–0.95) and test-retest (0.73–0.86) reliabilities are high.
Edinburgh Postnatal Depression Scale (EPDS) [23]	10-item screening measure for postnatal depression. Total scores range from 0 to 30, with higher scores indicating greater depressive symptoms. Using a score of ≥13 as cut-off, the scale has acceptable sensitivity (0.86), specificity (0.78), and positive predictive value (73%). Internal consistency reliability is high (0.87), and the scale is sensitive to changes in severity of depressive symptoms over time.
Parental Stressor Scale: Neonatal Intensive Care Unit (PSS:NICU) [24]	26-item scale that captures parental perceptions of stress in the NICU on 3 subscales: sights and sounds (6 items), infant behavior and appearance (13 items), and parental role alterations (7 items). A total score is calculated by averaging the responses on all items. The theoretical range of scores is 1 to 5; higher scores indicate greater overall stress. Internal consistency reliability is 0.89 for the total score.
Perceived Maternal Parenting Self-Efficacy (PMP S-E) tool [25]	20-item measure of parenting self-efficacy in mothers of hospitalized preterm infants. Measures maternal perceptions of their abilities across 4 subscales: care taking procedures (e.g., feeding and bathing), evoking behavior(s) (e.g., soothing the infant), reading behavior(s) or signalling (e.g., knowing when the infant is tired), and situational beliefs (e.g., believing the infant responds well to them). Total scores range from 20 to 80, with higher scores indicating greater perceived parenting self-efficacy. Internal consistency (0.91) and test-retest (0.96) reliabilities are high.

### Statistical analysis

After completion of the study, a statistical error was discovered in the sample size estimation, making it insufficient for the proposed cRCT design based on clustering using a random effects model (see published correction to trial protocol) [27]. With only 10 available clusters in the province, it was unfeasible to achieve adequate power to demonstrate a 10% difference between groups in LOS with the original analysis approach [28]. That is, the power reaches an asymptote of about 20% at a trial N of 2000 infants. Increasing the overall N beyond this would not increase the power of the trial, and therefore the original trial design was futile. An independent biostatistician identified that significant baseline variation in site and infant characteristics required adjustment to confirm differences in LOS.

The independent biostatistician developed an a priori risk-adjustment model that predicted LOS for each infant based on site geographic area (urban vs regional), GA in weeks, birth weight, mode of delivery, use of total parenteral nutrition, and neonatal hyperbilirubinemia. For each site, the observed LOS was compared to the predicted LOS. We then used a linear regression model to conduct a site-level analysis comparing the adjusted mean LOS for Alberta FICare™ versus standard care, weighted by number of participants at each site. Finally, we used a permutation test to confirm our test of significance, comparing our observed effect to the distribution of effects for all possible site allocations. We analyzed secondary infant outcomes using Pearson’s Chi-square tests. As with infant LOS, we used a risk-adjustment approach to assess maternal outcomes at discharge for each site, then conducted site-level analysis using linear regression. We controlled for mothers’ baseline scores by including them as covariates in the risk-adjustment model for each maternal outcome.

All analyses were by intention-to-treat and included all participants with available data, regardless of protocol deviation. For infants and their mothers who were transferred between intervention and standard care sites, the group of the original enrolling site was used in analyses. Analysis of maternal outcomes included all participants who completed both the admission and discharge surveys, irrespective of whether they were completed in the time frames specified by the protocol. The majority (80%) of admission surveys were completed within 72 h of admission (range day 1 to day 21), and 88% of discharge surveys were completed in the 72 h prior to discharge (range 15 days prior to 19 days post discharge). We excluded 6 discharge surveys that were completed over 7 days post discharge as they may not have accurately reflected maternal outcomes at discharge. All tests were two-sided, with significance set at ≤ .05. We did not correct for multiple comparisons because we had only one primary outcome. We used R version 3.6.2 (The R Foundation, Vienna, Austria) and SPSS version 25 (IBM Corp, Armonk, New York) for analysis.

## Results

**Fig. 1:**
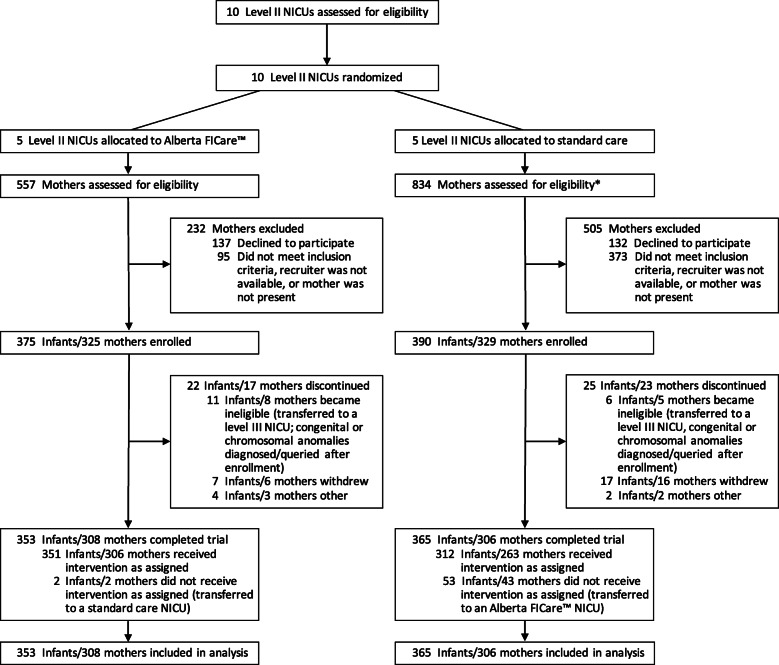
CONSORT flow diagram. *One large academic site allocated to standard care had dedicated research nurses who were extremely diligent in approaching every mother to assess eligibility and recording the number of mothers missed due to a recruiter not being available or mother not being present. Thus, a larger number of mothers were assessed and excluded in the standard care group compared to the Alberta FICare™ group. Abbreviation: *FICare* Family Integrated Care

Between December 15, 2015 and July 26, 2018, we enrolled 765 infants and 654 mothers; 375 infants/325 mothers at intervention sites and 390 infants/329 mothers at standard care sites (Fig. 1). An equal proportion of infants (6%) in both groups were excluded, most commonly due to becoming ineligible (e.g., transfer to a level III NICU) or their mothers withdrawing from the study. The final sample consisted of 353 infants/308 mothers in the intervention group and 365 infants/306 mothers in the standard care group. See Table 2 for infant and maternal characteristics. Compared to the standard care group, the intervention group had a significantly higher proportion of mothers who were Caucasian and born in Canada (χ^2^ *P* = .01 for both variables). However, in univariate regression analyses, neither of these maternal variables were associated with infant LOS (born in Canada vs not: *R*^2^ = .000, *P* = .74; Caucasian vs non-Caucasian: *R*^2^ = .002, *P* = .24).

**Table 2:** Infant and maternal characteristics

Characteristic	No.	Alberta FICare™	No.	Standard care
**Infant characteristics**		**(*n*** **= 353)**		**(*n*** **= 365)**
Singleton	353	263 (74.5)	365	247 (67.7)
Gestational age, wk	353		365	
32		74 (21.0)		63 (17.3)
33		101 (28.6)		97 (26.6)
34		178 (50.4)		205 (56.2)
Sex	353		365	
Male		190 (53.8)		195 (53.4)
Female		163 (46.2)		170 (46.6)
Birthweight, g, mean (SD)	353	2163 (395)	365	2120 (411)
Caesarean delivery	353	173 (49.0)	365	181 (49.6)
Apgar score < 7 at 1 min	351	78 (22.2)	364	110 (30.2)
Apgar score < 7 at 5 min	351	31 (8.8)	364	25 (6.9)
Infant of a mother with diabetes	341	57 (16.7)	359	61 (17.0)
Respiratory diagnoses^a^	353	217 (61.5)	356	225 (63.2)
Neonatal hyperbilirubinemia	353	294 (83.3)	364	270 (74.2)
Hypoglycemia	351	110 (31.3)	346	102 (29.5)
TPN required	352	154 (43.8)	358	179 (50.0)
IV fluids required	351	192 (54.7)	356	170 (47.8)
**Maternal characteristics**		**(*n*** **= 308)**		**(*n*** **= 306)**
Age, y, mean (SD)	303	30.7 (5.7)	295	31.3 (5.3)
Marital status	303		301	
Married or cohabiting		285 (94.1)		276 (91.7)
Single		15 (5.0)		21 (7.0)
Prefer not to answer/don’t know		3 (1.0)		4 (1.3)
Education	302		300	
High school diploma or less		72 (23.8)		58 (19.3)
Postsecondary certificate/diploma		75 (24.8)		77 (25.7)
College/university degree		155 (51.3)		165 (55.0)
Employment	302		300	
Employed/maternity leave		221 (73.2)		220 (73.3)
Homemaker/not in the labor force		50 (16.6)		48 (16.0)
Unemployed		7 (2.3)		7 (2.3)
Other		20 (6.6)		20 (6.7)
Prefer not to answer/don’t know		4 (1.3)		5 (1.7)
Annual family income	302		300	
< $40,000		18 (6.0)		27 (9.0)
$40,000 to $79,999		55 (18.2)		68 (22.7)
≥ $80,000		178 (58.9)		157 (52.3)
Prefer not to answer/don’t know		51 (16.9)		48 (16.0)
Race/ethnicity	299		297	
Caucasian		222 (74.2)		184 (62.0)
Indigenous		22 (7.4)		22 (7.4)
Asian (East, South, or Southeast)		35 (11.7)		50 (16.8)
Black		7 (2.3)		17 (5.7)
Other^b^		13 (4.3)		24 (8.1)
Born in Canada	301	243 (80.7)	299	215 (71.9)

Data are presented as n (%) unless otherwise indicated. No. varies due to missing data

*Abbreviations*: *FICare* Family Integrated Care, *TPN* total parenteral nutrition, *IV* intravenous

^a^ Includes apnea, transient tachypnea of the newborn, and respiratory distress syndrome

^b^ Includes Latin American, Middle Eastern, and Mixed

### Primary outcome

**Fig. 2:**
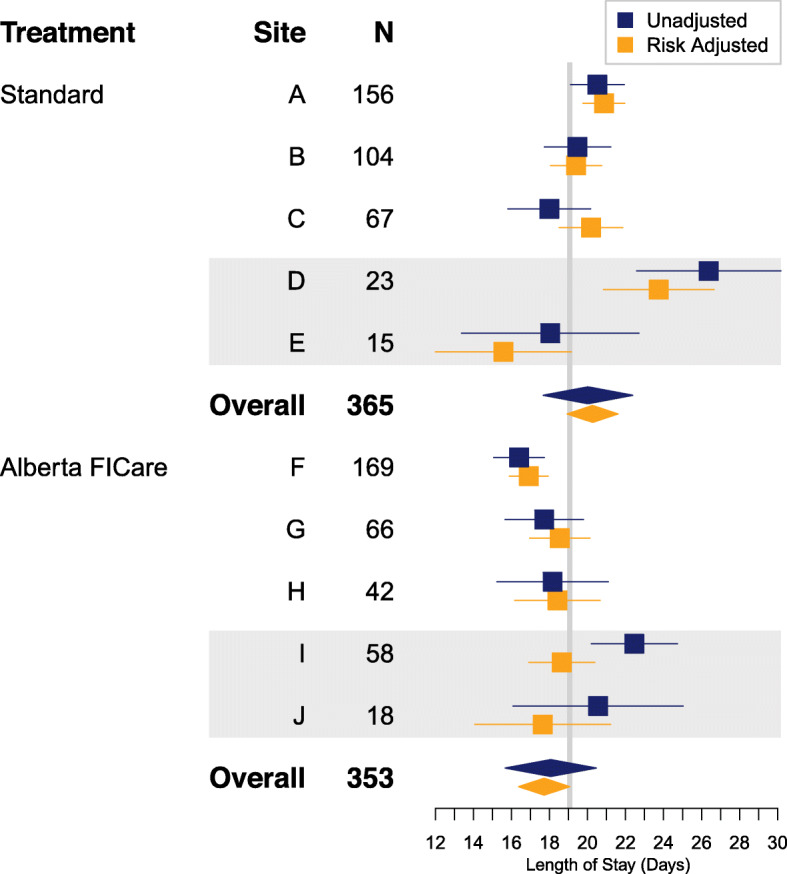
Forest plot of unadjusted and risk-adjusted mean infant LOS for each site. Horizontal lines represent 95% confidence intervals. Grey bars indicate regional sites. The risk-adjusted model included gestational age in weeks, birth weight, delivery mode, neonatal hyperbilirubinemia, total parenteral nutrition, and site geographic area. Abbreviations: *FICare* Family Integrated Care, *LOS* length of stay

Mean LOS adjusted for site geographic area and infant risk factors was shorter for infants in the Alberta FICare™ group (17.62 days; 95% CI, 16.27 to 18.96) than the standard care group (20.16 days; 95% CI, 18.83 to 21.50), with a treatment effect of − 2.55 days in favor of Alberta FICare™ (95% CI, − 4.46 to − 0.66; *P* = .02; Table 3). A permutation test confirmed this result with *P* = .05. The forest plot in Fig. 2 summarizes the unadjusted and geographic area/risk-adjusted mean LOS for each site.

**Table 3:** Infant outcomes

Outcome	Alberta FICare™ (*n* = 353)	Standard care (*n* = 365)	Effect	*P* value	Permutation test *P* value
**Primary outcome**	**Mean (95% CI)**	**Mean (95% CI)**	**Mean difference (95% CI)**		
Length of stay, days					
Unadjusted	17.62 (15.23, 20.01)	19.58 (17.23, 21.94)	−1.96 (−5.32, 1.40)	.22	.28
Adjusted for site geographic area	18.06 (16.55, 19.58)	20.47 (18.98, 21.96)	−2.40 (− 4.53, − 0.28)	.03	.13
Adjusted for site geographic area/ infant risk factors	17.62 (16.27, 18.96)	20.16 (18.83, 21.50)	−2.55 (− 4.44, − 0.66)	.02	.05
**Secondary outcomes**	***n*** **(%)**	***n*** **(%)**	**OR (95% CI)**		
Hospital readmission to 2 months CA	22 (6.2)	24 (6.6)	0.94 (0.52, 1.72)	.85	–
Emergency department visit to 2 months CA	83 (23.5)	93 (25.5)	0.90 (0.64, 1.26)	.54	–
Feeding at discharge^a^					
Breastmilk only	217 (70.0)	175 (66.8)	–	.57	–
Combination	78 (25.2)	76 (29.0)			
Formula only	15 (4.8)	11 (4.2)			

*Abbreviations*: *FICare* Family Integrated Care, *CA* corrected age

^a^ *n* = 310 for Alberta FICare group and *n* = 262 for standard care group due to missing data

### Secondary outcomes

Groups were similar in proportions of infants with hospital readmissions (6.2% Alberta FICare™ vs 6.6% standard care; *P* = .85) and emergency department visits (23.5% Alberta FICare™ vs 25.5% standard care; *P* = .54) to 2 months CA (Table 3). Type of feeding and maternal outcomes at discharge were also not significantly different between groups; see Tables 3 and 4, respectively.

**Table 4:** Maternal outcomes

	Baseline		Discharge				
Outcome measure	Alberta FICare™ Mean (*SD*)	Standard care Mean (*SD*)	Alberta FICare™ Mean (*SD*)	Standard care Mean (*SD*)	Model adjustment	Mean difference at discharge (95% CI)	*P* value
STAI–Trait^a^	35.43 (9.36)	34.85 (8.91)	–	–		–	–
STAI–State^a^	38.24 (12.01)	37.22 (11.36)	31.26 (10.00)	32.62 (9.94)	None	−1.80 (−4.74, 1.13)	.20
					Geographic area	−1.61 (−4.36, 1.13)	.21
					Geographic area and infant risk factors	−1.43 (−4.23, 1.36)	.27
EPDS^b^	7.85 (4.76)	8.01 (4.87)	6.20 (4.34)	7.03 (4.62)	None	−0.75 (−1.80, 0.30)	.14
					Geographic area	−0.70 (−1.75, 0.36)	.17
					Geographic area and infant risk factors	−0.61 (−1.66, 0.45)	.22
PSS:NICU^c^	2.59 (0.80)	2.61 (0.82)	2.43 (0.84)	2.57 (0.85)	None	−0.13 (− 0.28, 0.02)	.09
					Geographic area	−0.12 (− 0.27, 0.03)	.10
					Geographic area and infant risk factors	−0.13 (− 0.29, 0.04)	.11
PMP S-E^d^	62.76 (10.71)	63.53 (10.66)	73.64 (6.25)	72.35 (6.64)	None	1.45 (−0.81, 3.71)	.16
					Geographic area	1.11 (−0.46, 2.68)	.14
					Geographic area and infant risk factors	0.96 (−0.57, 2.50)	.19

All models included admission scores as covariates. The models for state anxiety also controlled for trait anxiety, which was measured only at admission

*Abbreviations*: *FICare* Family Integrated Care, *STAI* State-Trait Anxiety Inventory, *EPDS* Edinburgh Postnatal Depression Scale, *PSS:NICU* Parental Stressor Scale: Neonatal Intensive Care Unit, *PMP S-E* Perceived Maternal Parenting Self-Efficacy

^a^ *n* = 267 for Alberta FICare™ group and *n* = 215 for standard care group

^b^ *n* = 268 for Alberta FICare™ group and n = 215 for standard care group

^c^ *n* = 268 for Alberta FICare™ group and n = 217 for standard care group

^d^ *n* = 266 for Alberta FICare™ group and *n* = 217 for standard care group

## Discussion

In our cRCT in level II NICUs, adjusting for site geographic area and infant risk factors, preterm infants in the Alberta FICare**™** group were discharged home 2.55 days sooner than infants in the standard care group. This decrease in LOS, which was greater than our hypothesized 10%, is an important positive outcome for the health system and families. Feeding type at discharge and proportions of infants who were readmitted to hospital or had an emergency department visit to 2 months CA were similar in both groups. Contrary to our hypothesis, we did not find significant group differences on maternal outcomes; however, all effects demonstrated a tendency to favor the Alberta FICare™ group.

The reduction in hospital LOS with Alberta FICare™ contrasts with a meta-analysis of 19 RCTs of FCC interventions for preterm infants and parents that found no difference in LOS [29]. There may be several reasons for these differences, including considerable variability in components of the FCC interventions (e.g., educational, psychosocial, NICU environment), context of NICU care (e.g., Asia, Europe, Australia/New Zealand, and North America), and GA of infants (23–42 weeks). It may be that multicomponent interventions, such as FICare, may be required to demonstrate differences in LOS. However, the FICare level III NICU cRCT did not demonstrate a group difference in LOS [16].

Differences in results regarding LOS between our study and the FICare level III NICU cRCT may be attributed to differences in the FICare models. In contrast to the FICare level III NICU model where staff education and support were considered key components of the model, in the Alberta FICare™ trial these were considered part of the implementation process. In our study, we monitored intervention fidelity and provided booster doses of training, which is essential to ensuring interventions are delivered as intended [30]. In contrast to the FICare level III NICU model [15, 16] where parents typically received a printed orientation package and attended facilitated group education sessions, in Alberta FICare™ Parent Education used a multimodal approach including an evidence-informed Parent Education Pathway that HCP signed off as parents acquired knowledge and skills. Parent education was supplemented by the free Life’s Little Love app that parents could download on their personal devices. The FICare level III NICU model [15, 16] emphasized creating a physical environment where parents could stay at the bedside, while in Alberta FICare™ greater emphasis was placed on creating a unit culture where parents felt welcomed and included in the care team. Unlike the FICare level III cRCT [16], we screened for postnatal depression and referred mothers in both groups to existing social work services. To signal the importance of parental presence in the NICU as a precursor to parental participation in care, we provided parking passes to families in the intervention group for the duration of hospitalization. Many mothers and some fathers recorded their thoughts and feelings in the investigator-designed parent journals, which may have been therapeutic [31].

Integrating families into the care of their infant in NICU requires a shift in culture where parents are educated and supported by HCP to provide care for their infant from the time of admission. As parents become more confident and assume greater responsibility for the care of their infant(s) in the NICU, it is critical for safe delivery of care to have clearly defined roles for parents and HCP. Thus, the Relational Communication component of Alberta FICare™ may be an important component to prevent assumptions about the family’s situation and their readiness and willingness to provide care, and understand patterns of interactions that enable integration of families into the care team [19].

Rates of any breastmilk feeding at discharge were high in both groups, with 95 and 96% of infants in the intervention and standard care group, respectively, receiving breastmilk only or combination of breastmilk and formula. These rates were higher than those reported in the FICare level III NICU cRCT [16], where the rate of any breastmilk feeding at discharge was 75% in the FICare group and 81% in the standard care group (*P* = .004). Without data specific to infants in NICU, we speculate that failure to find group differences in our study may be attributed to high rates of breastfeeding initiation in Alberta [32].

Although effects favored Alberta FICare™, we found no significant group differences in maternal anxiety, depressive symptoms, and stress, which contrasts with the results of the FICare level III NICU cRCT [16] and a meta-review of diverse interventions for parents of preterm infants [33]. The meta-review did not include FICare interventions, and most effective interventions included a home visitation component [33]. In the FICare level III NICU cRCT, investigators measured differences in maternal outcomes within a 21-day window [16]. In the Alberta FICare™ cRCT, we measured maternal outcomes between admission and discharge. If parental ability to provide care at home is a criterion for discharge, then we speculate our lack of group difference may be related to timing of measurement rather than the effect of Alberta FICare™. Our results are similar to Ingram and colleagues who did not find any change in parenting self-efficacy in their before and after study of family-centred neonatal discharge planning with infants born at 27–33 weeks GA [34].

As hypothesized, we did not find group differences in proportions of infants requiring hospital readmissions and emergency department visits. Our result related to readmission is contrary to the results of a meta-analysis of 4 RCTs (3 from China, 1 from Iran) of FCC interventions in NICU that reported significantly lower readmission rates in the FCC group versus the standard care group [29]. The studies included in the meta-analysis reported small sample sizes (range 46–130), < 10 readmissions in both groups across studies, and wide range of infant GAs [29]. We speculate that differences in results between our cRCT and the meta-analysis [29] may be attributed to small samples and different GAs. To our knowledge, ours is the first cRCT to report emergency department visits as an outcome related to FICare interventions.

Our study has several strengths. First, the theoretical underpinnings of Alberta FICare™ will enable a better understanding of the potential mechanisms underlying its effect on outcomes. Future research is needed to identify components that make the greatest contribution to effectiveness and should be implemented in resource constrained settings. Second, our cRCT was designed and implemented with input from key stakeholders; parents were included in design and training for HCP at intervention sites. Co-design strengthens the relevance and sustainability of intervention [35]. Third, all available level II NICUs in Alberta participated and completed the study. Finally, quarterly site visits to monitor intervention fidelity increased confidence that Alberta FICare™ was implemented as designed, and the cRCT design minimized contamination across sites.

This study has several limitations. First, components of the Alberta FICare™ intervention (required time spent in NICU and parking passes) were intentionally designed to facilitate parental presence. Although parental presence may have increased parental confidence and shortened LOS, these intervention components were designed as part of the Alberta FICare™ bundle; effects of individual components cannot be disentangled from the whole. Health system policy changes that provide parking passes to all parents of infants in NICU and repeated evaluation of Alberta FICare™ may identify the most effective components of the intervention. Although there was a statistically significant group difference in parental time spent in NICU (9.00 vs. 7.79 h for Alberta FICare™ and standard care groups, respectively), a 1.21-h difference is unlikely to be clinically relevant. Second, the rate of missing maternal data differed between groups; 87% of mothers in the intervention group completed both surveys versus 71% of mothers in the standard care group. Since data on type of feeding was reported by mothers on the discharge survey, this resulted in more missing infant feeding data for infants in the standard care group compared to infants in the Alberta FICare™ group. Third, Alberta has a single health services delivery system with many standardized structures and processes. Thus, results may not be generalizable to other systems of care. Fourth, there were insufficient sites in the province for a cRCT and this study was underpowered for the planned analysis.

## Conclusions

Alberta FICare™ in level II NICUs reduced hospital LOS in preterm infants born between 32^0/7^ and 34^6/7^ weeks GA, without concomitant increases in readmissions and emergency department visits. A small number of sites in a single jurisdiction and inclusion of a select group of mothers and infants limit generalizability of findings. Country-wide replication of the trial in all level II NICUs would increase generalizability.

## Data Availability

The study protocol is publicly and freely available [17]. Data collected for the study will be made available as individual de-identified participant data and as metadata with an accompanying data dictionary in PolicyWise Secondary Analysis to Generate Evidence (SAGE; https://policywise.com/sage/), a secure data repository that provides technical infrastructure and governance processes that protect participant privacy and ensure ethical re-use of data by approved researchers only. Data will be made available indefinitely in SAGE following publication of all study findings.

## Abbreviations

cRCT: Cluster randomized controlled trial
CA: Corrected age
EPDS: Edinburgh Postnatal Depression Scale
FCC: Family Centred Care
FICare: Family Integrated Care
GA: Gestational age
HCP: Health care providers
LOS: Length of stay
NICU: Neonatal intensive care unit
PMP S-E: Perceived Maternal Parenting Self-Efficacy
PSS:NICU: Parental Stressor Scale: Neonatal Intensive Care Unit
STAI: State-Trait Anxiety Inventory
